# Supplementary material for: A Comparative Analysis of Orthotopic and Subcutaneous Pancreatic Tumour Models: Tumour Microenvironment and Drug Delivery
Source: Cancers (Basel). 2023 Nov 14;15(22):5415. doi: 10.3390/cancers15225415 (PMC10670202; doi:10.3390/cancers15225415)
Supplement: Supplementary file 1 [file cancers-15-05415-s001.zip › cancers-2668061-supplementary.pdf]

Supplementary materials.

Masson trichrome (MT) images of orthotopic and subcutaneous KPC tumours are shown in Figure S1, and five images are presented per group. The orthotopic tumours (Figure S1, a) showed several small focal areas with tumour necroses, glandular and ductal structures, and infiltration in adjacent soft tissues, including skeletal muscle. The subcutaneous tumours (Figure S1, b) had large necrotic areas and infiltration in the skeletal muscle in most of the tumours.

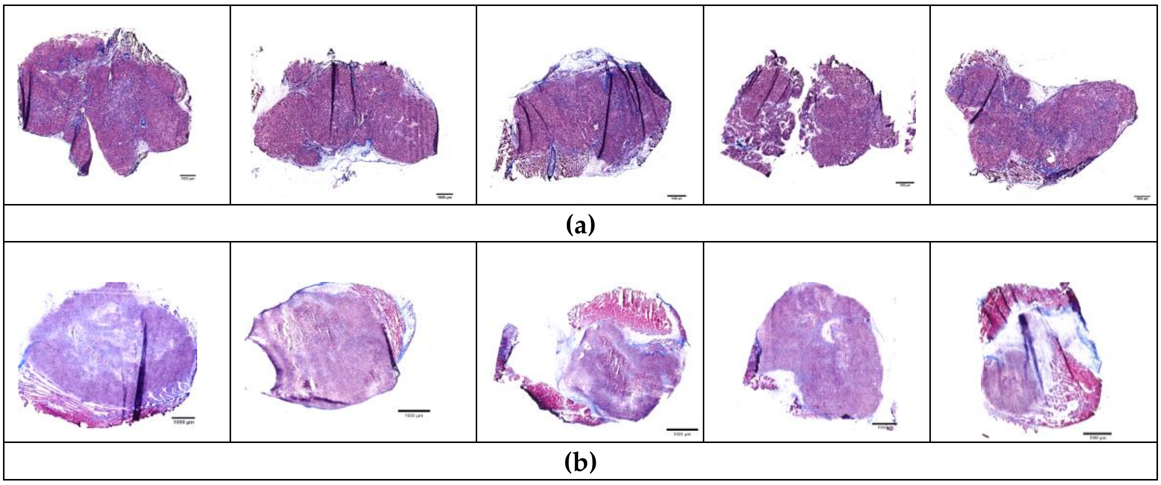

Figure S1. Masson trichrome (MT) images of orthotopic (a) and subcutaneous (b) KPC tumours. Collagen is stained in blue. The scale bar represents 1000 μm.

B-mode images and ROIs of orthotopic and subcutaneous KPC tumours are shown in Figure S2, and five images are presented per group. The orthotopic tumours (Figure S2, a) showed tumours infiltrating through the abdominal wall and protruding out towards the skin.

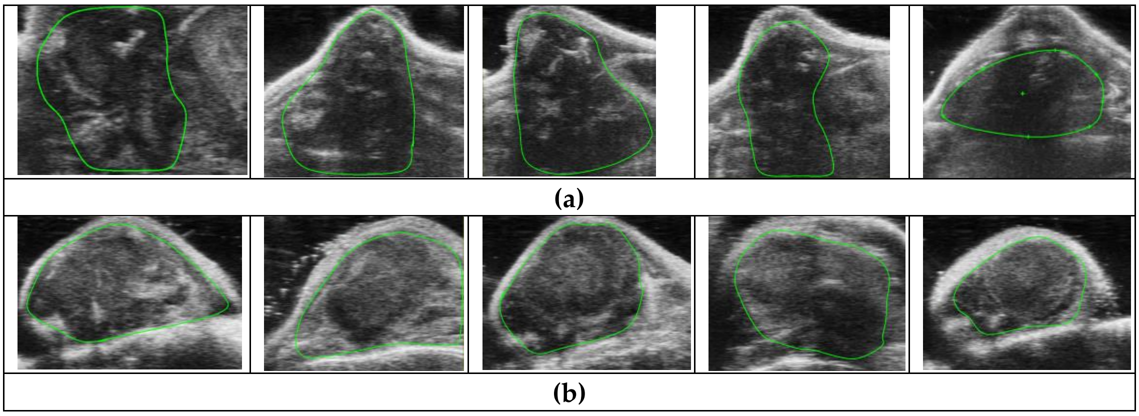

Figure S2. B-mode ultrasound images and ROIs of orthotopic (a) and subcutaneous (b) KPC tumours.

Second harmonic imaging microscopy (SHIM) images of orthotopic and subcutaneous KPC tumours are shown in Figure S3, and five images are presented per group. The orthotopic tumours (Figure S3, a) showed several potential ductal structures surrounded with collagen. The subcutaneous tumours (Figure S3, b) presented more aligned fibres.

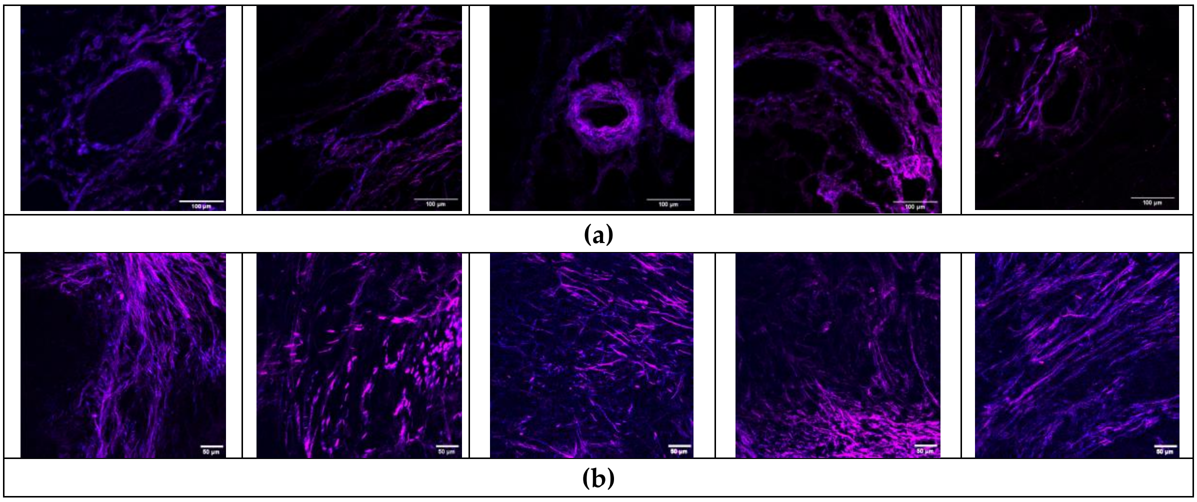

Figure S3. Second-harmonic imaging microscopy (SHIM) images of orthotopic (a) and subcutaneous (b) KPC tumours. Blue represents the forward second harmonic generated (SHG) collagen signal and magenta for the matching backward signal.

Confocal laser scanning microscopy (CLSM) images of functional vessels of orthotopic and subcutaneous KPC tumours are shown in Figure S4, and five images are presented per group. Both orthotopic tumours (Figure S4, a) and subcutaneous tumours (Figure S4, b) presented substantial variability in the number of functional vessels, as shown in the images. Images exhibiting a higher density of functional vessels are positioned on the left side.

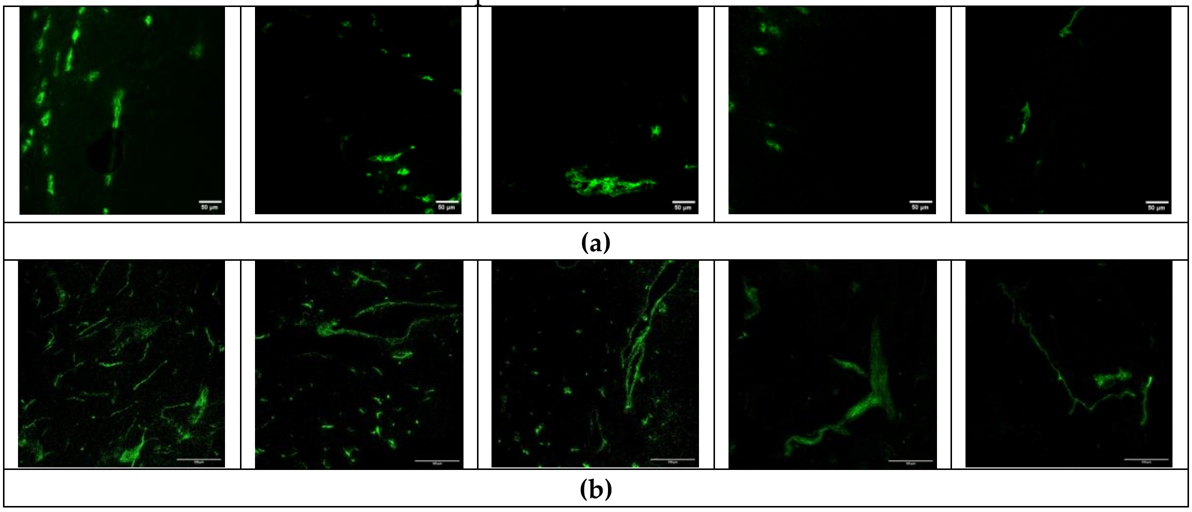

Figure S4. Confocal laser scanning microscopy (CLSM) of lectin-FITC-labelled vessels of orthotopic (a) and subcutaneous (b) KPC tumours.
